# Wide FOV metalens for near-infrared capsule endoscopy: advancing compact medical imaging

**DOI:** 10.1515/nanoph-2024-0393

**Published:** 2024-10-17

**Authors:** Mojtaba Moghaddasi, Erik Edilson Perez Coca, Danni Ye, Diego Alejandro Flores, Xudong Wu, Abdul Jalal, Ziming Ren, Fahimeh Abrinaei, Bin Hu

**Affiliations:** National Key Laboratory on Near-Surface Detection, School of Optics and Photonics, Beijing Institute of Technology, Beijing 100081, China; School of Mechatronical Engineering, Beijing Institute of Technology, Beijing 100081, China; School of Chinese Materia Medica, Beijing University of Chinese Medicine, Beijing 102488, China; School of Computer Science and Technology, 47833 Beijing Institute of Technology, Beijing 100081, China; Department of Physics, Central Tehran Branch, Islamic Azad University, Tehran, Iran; Department of Physics, East Tehran Branch, Islamic Azad University, Tehran, Iran

**Keywords:** capsule endoscopy, metalens, metasurface, nano-optics, narrow band imaging, wide field of view

## Abstract

This study presents the design, fabrication, and characterization of a wide field-of-view (FOV) metalens optimized for capsule endoscopy. The metalens achieved a 165° FOV with a high modulation transfer function (MTF) of 300 lines per millimeter (lp/mm) across the entire FOV, operating in the near-infrared (NIR) narrow-bandpass imaging at 940 nm. The performance of the metalens-based system is evaluated using two bandwidths, 12 nm and 32 nm, showing MTF values of 0.2 and 0.3 at 250 lp/mm, respectively. The metalens-based system maintains a compact form factor with a total track length of 1.4 mm and a diameter of 1.58 mm. Compared to a traditional 108° FOV endoscope, the nano-optic capsule endoscope demonstrated superior performance in terms of FOV, contrast, and resolution. This advancement represents a significant step toward enhancing diagnostic capabilities in medical imaging, offering improved performance in a more compact package compared to conventional optics.

## 1 Introduction

Optical elements are essential components in numerous modern imaging systems, ranging from tiny lenses in smartphones to sophisticated optics used in medical devices. Metalenses as new and flat optical elements represent a significant technological advancement over traditional lenses, offering improved performance, reduced size and weight, and increased flexibility in design and fabrication [1], [2], [3]. Traditional lenses rely on refraction to focus light, requiring a curved surface to bend the light rays. In contrast, metalenses utilize phase manipulation via nanostructured metasurfaces to achieve the desired focusing effect. This unique approach to light manipulation allows metalenses to be fabricated with a significantly reduced thickness compared to their traditional lens counterparts. Despite their thin profile, metalenses can still deliver comparable or even superior optical performance in terms of focusing, aberration correction, and other key parameters [4], [5].

Metalenses comprise arrays of subwavelength nanostructures, known as meta-atoms, which locally and coherently modulate the phase of incident light across the entire metasurface, creating the necessary phase profile for focusing [6]. This unique capability enables the creation of ultra-thin, flat optical components that can achieve complex optical functions, opening new possibilities for compact and lightweight optical systems in various applications, including imaging, sensing, and display technologies [7], [8], [9], [10], [11].

Wide FOV metalenses have garnered particular interest due to their potential to provide aberration-free, polarization-free, and high-quality imaging over large angular ranges. Their advantages make them suitable for various applications such as imaging, display, sensing, and beam steering [12], [13], [14], [15]. Recent research has demonstrated significant progress in this area, with achievements including FOVs up to 178° for circularly polarized light [16], full-color imaging with a 100° FOV using quadratic metalenses [17], and broadband achromatic design in the mid-infrared [18]. A Huygens metalens, designed for outdoor photographic and surveillance applications in the NIR region, obtained a 30° FOV [19]. A near 180° angular FOV of Huygens metalenses in the NIR and mid-infrared regions has also been proposed [20]. Additionally, metalenses operating at a wavelength of 532 nm arranged in a hexagonal pattern have been designed [21]. Recently, a long-wave infrared metalens with a 140° FOV and a diameter of about 4 cm was reported [22].

Despite these advances, the application of wide FOV metalenses in medical imaging, particularly in capsule endoscopy, remains largely unexplored. In the field of imaging, wide FOV metalenses can be used in various imaging applications, including surveillance, medical imaging, and virtual and augmented reality. Endoscopy is a crucial tool in modern medical imaging, allowing for minimally invasive inspections, early disease detection, precise guidance during therapeutic procedures, and versatile use across different parts of the body [23], [24]. As technology advances, endoscopy is expected to play an increasingly significant role in enhancing patient care and outcomes [25]. Incorporating metalenses into optical imaging endoscopes provides substantial benefits, including enhanced miniaturization, improved performance, greater simplicity, increased flexibility, and reduced costs [26], [27], [28]. These benefits improve diagnostic capabilities, enhanced patient care, and more efficient medical imaging procedures [29]. Capsule endoscopes offer several advantages over fiber endoscopes, including noninvasiveness, no sedation required, wider imaging range, improved patient comfort, increased versatility, reduced complications, increased accessibility, improved diagnostic accuracy, cost-effectiveness, and future advancements [7], [30], [31], [32]. However, the effectiveness of capsule endoscopes depends on the quality of imaging, which is often constrained by the size limitations of traditional optical components. The advent of metalenses presents a groundbreaking solution to this challenge. Metalenses offer high-resolution imaging in a compact form factor and can be seamlessly integrated with other optical devices. This promises improved diagnostic accuracy and enables multifunctional capabilities in next-generation medical imaging devices. The narrow-band imaging (NBI) is utilized because it offers high-performance imaging due to lacking the chromatic aberration in metalens. NBI has been applied in endoscopy systems for decades and can be widely used in the early detection of various diseases related to mucosal lesions, such as those in the upper and lower gastrointestinal tracts [33]. The advantages of NBI include being minimally invasive and intuitive, allowing for clear visualization of the surface capillary vessels. This enables us to observe endoscopic features, classify different organs, and enhance the diagnostic precision of tumors. Some studies have reported that NBI technology had a higher adenoma detection rate than white light endoscopy (WLE) [34], [35]. Furthermore, magnifying endoscopy with NBI proves effective in distinguishing intramucosal carcinoma from adenoma, thereby reducing cancer underdiagnosis. The detection rate for superficial esophageal squamous cell carcinoma, including high-grade intraepithelial neoplasia, is notably higher with NBI than with WLE [36].

This paper explores the potential of metalenses in capsule endoscopy, highlighting their role in revolutionizing imaging quality, device miniaturization, and functional diversity. We present the design, fabrication, and characterization of a 165° FOV metalens optimized for nano-optic capsule endoscopy devices using a narrow-bandpass spectrum centered at a wavelength of 940 nm. A wavelength of 940 nm was selected as a source due to its superior tissue penetration compared to the visible range. NBI systems presented comparable performance to white light imaging and chromoendoscopy systems, which can help diagnose more precisely [35], [36], [37], [38], [39].

We experimentally analyzed the effects of source bandwidth (12 nm and 32 nm) on full width at half maximum (FWHM), Strehl ratio (SR), point spread function (PSF), and modulation transfer function (MTF) of fabricated metalens. The performance of nano-optic capsule endoscope is compared with traditional bulky lens systems. The design concept, fabrication method, and characterization results are detailed in subsequent sections.

## 2 Design concept

**Figure 1:**
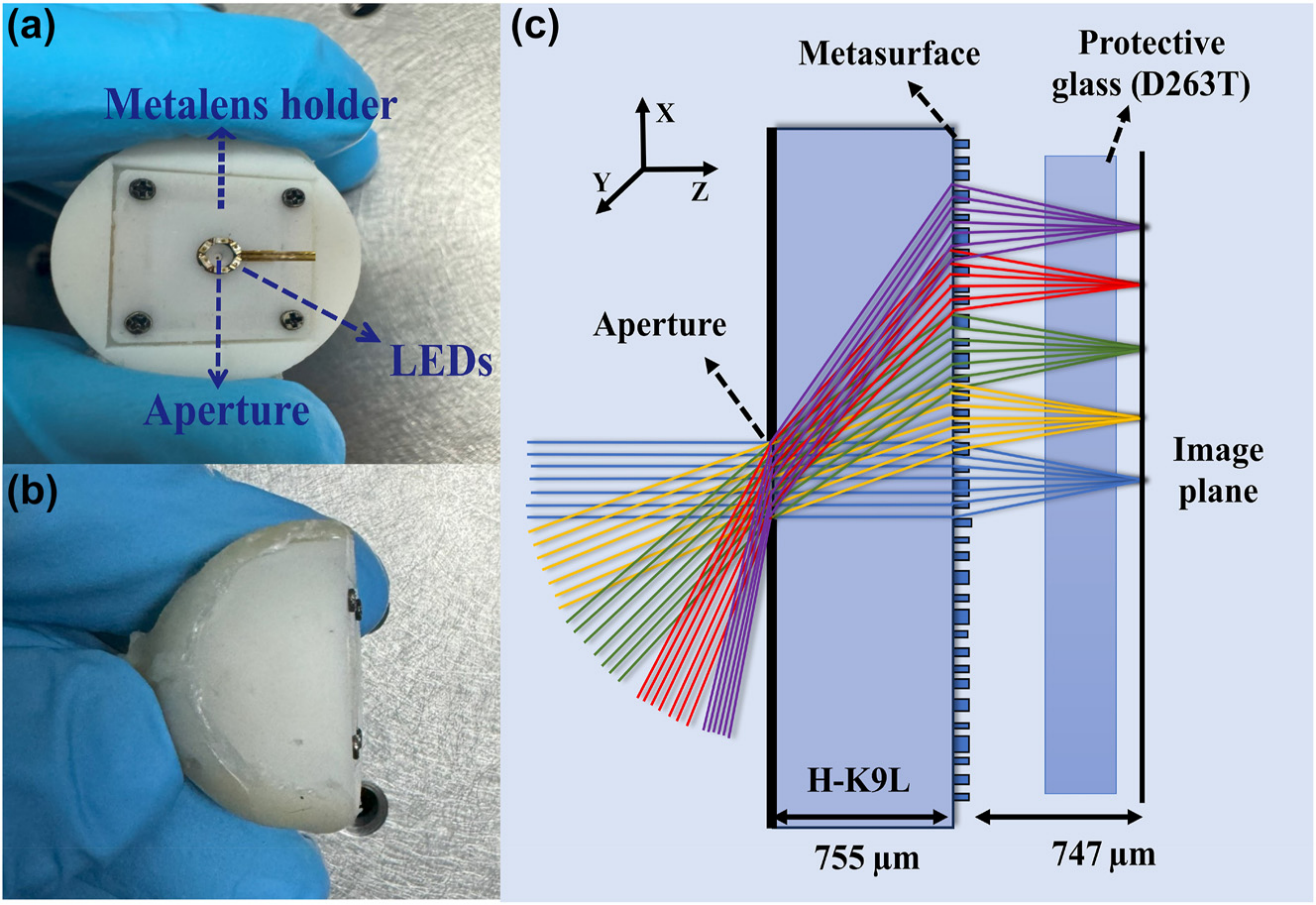
Metalens-based capsule endoscopy. (a) Front and (b) top view of the nano-optic capsule endoscopy system. (c) Ray tracing of different FOVs through the capsule endoscopy camera.

The design of an effective capsule endoscopy system requires careful consideration of several critical factors, including image quality, FOV, and form factor. In this study, we aimed to achieve an optical imaging component that is as compact as possible, featuring a short total track length and a small diameter of metalens. Nano-optic capsule endoscopy includes a metalens, a bandpass filter, a 940 nm commercial light-emitting diode (LED), an optical diffuser, an optical aperture, and a complementary metal-oxide semiconductor (CMOS) sensor. The design parameters are detailed in Table 1. Figure 1(a) and (b) presents the prototype of our nano-optic capsule endoscopy device. To achieve the FOV of 165°, we employed an image-space telecentric configuration. Figure 1(c) depicts the tangential ray trace of the designed metalens across 0–82.5°. The substrate material used was H-K9L, and a 260 µm aperture was implemented.

**Table 1:** Optical design parameters.

Items	Specifications
Center wavelength (nm)	940
Aperture diameter (mm)	0.26
FOV (°)	165
Working F-number	2.34
Focal length (mm)	0.605
Working distance (mm)	>15
Semidiameter metalens (mm)	<0.79
Total track length (mm)	<1.4
Designed MTF (%)	>20 at 300 lp/mm

For image capture, we selected the OV9734 CMOS sensor from Omnivision Co., which features a compact package and a 1,280 × 720 pixel array with a 1.4 µm pixel size. This sensor was chosen for its balance of resolution, size, and compatibility with our optical design.

### 2.1 Metalens design

The metalens was designed assisted by Zemax OpticStudio and Lumerical finite-difference time-domain (FDTD) commercial software. Forward design is used for meta-atom engineering, but other inverse design approaches, such as end-to-end methods [40], [41], [42], can also be employed. These approaches can help preserve full color in nano-optic capsule endoscopes for WLE. The optimized phase distribution was first obtained using Zemax OpticStudio, and then full-wave electromagnetic simulations were conducted with the Lumerical FDTD solver to design the nanostructures. The phase distribution across the diffractive surface was characterized using radially symmetric polynomials in cylindrical coordinates, as follows:

(1) $$\varphi \left(x,y\right)=M\overset{n}{\underset{i=1}{\sum }}a_{n}{\left(\frac{\rho }{R}\right)}^{2i}$$

In Equation (1), *R* represents the normalized radius of the designed metalens, *M* denotes the diffraction order, and *ρ* signifies the radius along the plane of the metalens. The coefficients *a*_*n*_ were treated as variables to minimize the spot size at the focal point for incident rays at various angles. Through our analysis, 10 terms of the polynomial provided adequate control over the phase, facilitating a high-quality design. The H-K9L glass was selected as a substrate with a thickness of 755 μm. One side of the substrate featured a circular aperture with a diameter of 260 µm, while the opposite side functioned as a metasurface. The Zemax OpticStudio analysis, which includes spot diagrams, as well as sagittal and tangential MTF diagrams, is discussed in Supporting Information S1.

**Figure 2:**
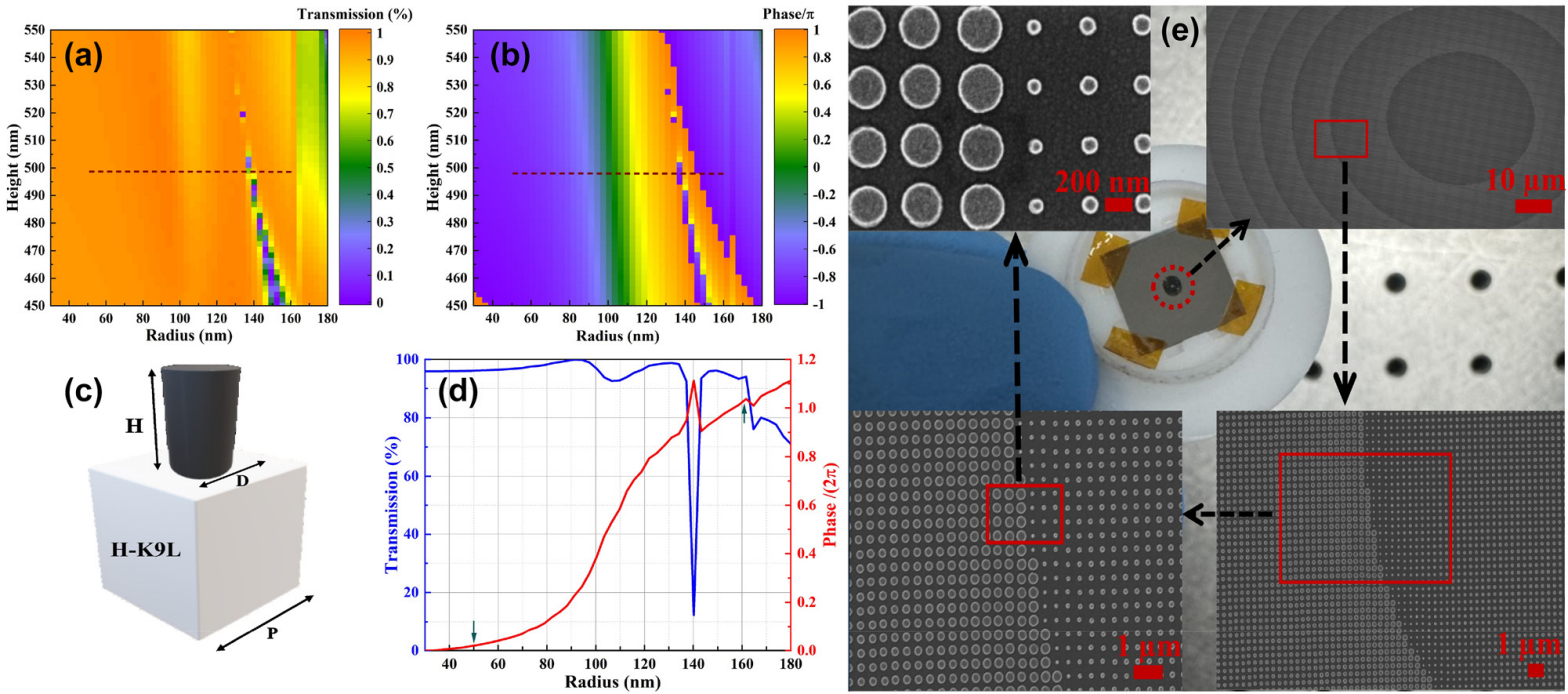
Design, simulation, and SEM visualization of fabricated metalens. (a) Transmission and (b) phase diagrams versus different heights and radii at a wavelength of ***λ*** = 940 nm. The dashed lines show the selected height and radii of nano-pillars. (c) The unit cell structure of the metalens. The nano-pillars are designed in a square lattice with a unit cell period of *P* = 400 nm. The imparted phase by a nano-pillar is managed by its diameter (*D* = 100–324 nm). (d) Transmission and phase diagram versus radius at 490 nm nano-pillar height. (e) Fabricated metalens and SEM images.

To achieve a polarization-independent structure, circular nano-pillars arranged in a cubic lattice were employed. The metalens is fabricated by positioning an array of circular amorphous silicon (a-Si) meta-atoms on an H-K9L substrate, with a schematic of the meta-atom depicted in Figure 2(c).

The a-Si is chosen for its high refractive index in the NIR range, while the H-K9L substrate is selected for its low refractive index and cost-effectiveness. The propagation phase can be controlled by adjusting the diameters of the meta-atoms. To identify high-performance nano-pillars, simulations were conducted varying the height, radius, and periodicity of the nano-pillars. As illustrated in Figure 2(a) and (b), a fixed nano-pillar height of 490 nm and radii ranging from 50 to 162 nm can achieve a 2*π* phase shift while maintaining transmission above 90 %. The dashed line shows the selected height and radii of nano-pillars; it covers 2*π* phase change and high transmission. Although there is a minor dip in performance at a radius of 140 nm, it does not significantly affect the overall efficiency of the metalens. Metalens parameters are specified in Table 2. The far-field Lumerical FDTD analysis was conducted to characterize the performance of the metalens. The focus spot distribution, PSF, and MTF, which indicate a diffraction-limited design, are discussed in Supporting Information S2.

**Table 2:** Metalens specification.

Items	Specifications
Metalens diameter (mm)	1.6
Nano-pillar height (nm)	490
Nano-pillar diameter (nm)	100–324
Nano-pillar aspect ratio	4.9:1
Period (nm)	400
Substrate thickness (μm)	755
Substrate material	H-K9L
Nano-pillar material	a-Silicon

### 2.2 Device fabrication and assembling

The optical components of the capsule endoscope include several essential parts: a 940 nm LED, a narrow-bandpass filter, a metalens, an optical aperture, and an OV9734 CMOS sensor. Commercial low-cost 940 nm LEDs typically have a bandwidth of approximately 60 nm. To reduce this bandwidth and minimize chromatic aberrations, a 12 nm commercial bandpass filter is employed. The OV9734 CMOS sensor is well-suited for capsule endoscopy applications due to its compact packaging, low power consumption, and dedicated infrared solution.

To elucidate the practical viability of this concept, we integrate the metalens with a CMOS sensor in a cohesive assembly. The mechanical mounts and housing of the device are fabricated using a 3D printer (Form 3) from Formlabs, allowing for rapid prototyping and iteration of the design. The integration of the metalens with the CMOS sensor posed a significant challenge due to the precise alignment of the spatial interval between the metalens and the CMOS imaging device. We addressed this using a custom assembling procedure involving four screws and springs for fine adjustment (Figure 1(a)).

The metalens was fabricated utilizing an electron beam lithography (EBL) technique. Initially, a 200 nm-thick layer of resist (AR-P-6200) was spin-coated onto a 490 nm-thick amorphous silicon-on-HK9L substrate and patterned using electron beam lithography (EBL). Following this, a chromium hard mask approximately 50 nm thick was deposited via electron-beam evaporation, and then the resist was removed through lift-off in N-methyl pyrrolidone at 120 °C for 2 h. Finally, the amorphous silicon layer was etched using a fluorine-based inductively coupled plasma etching process. The scanning electron microscopy (SEM) images of the fabricated metalens are shown in Figure 2(e). The following sections will detail the characterization and performance of this system, demonstrating its potential for capsule endoscopy application.

## 3 Characterization and discussion

**Figure 3:**
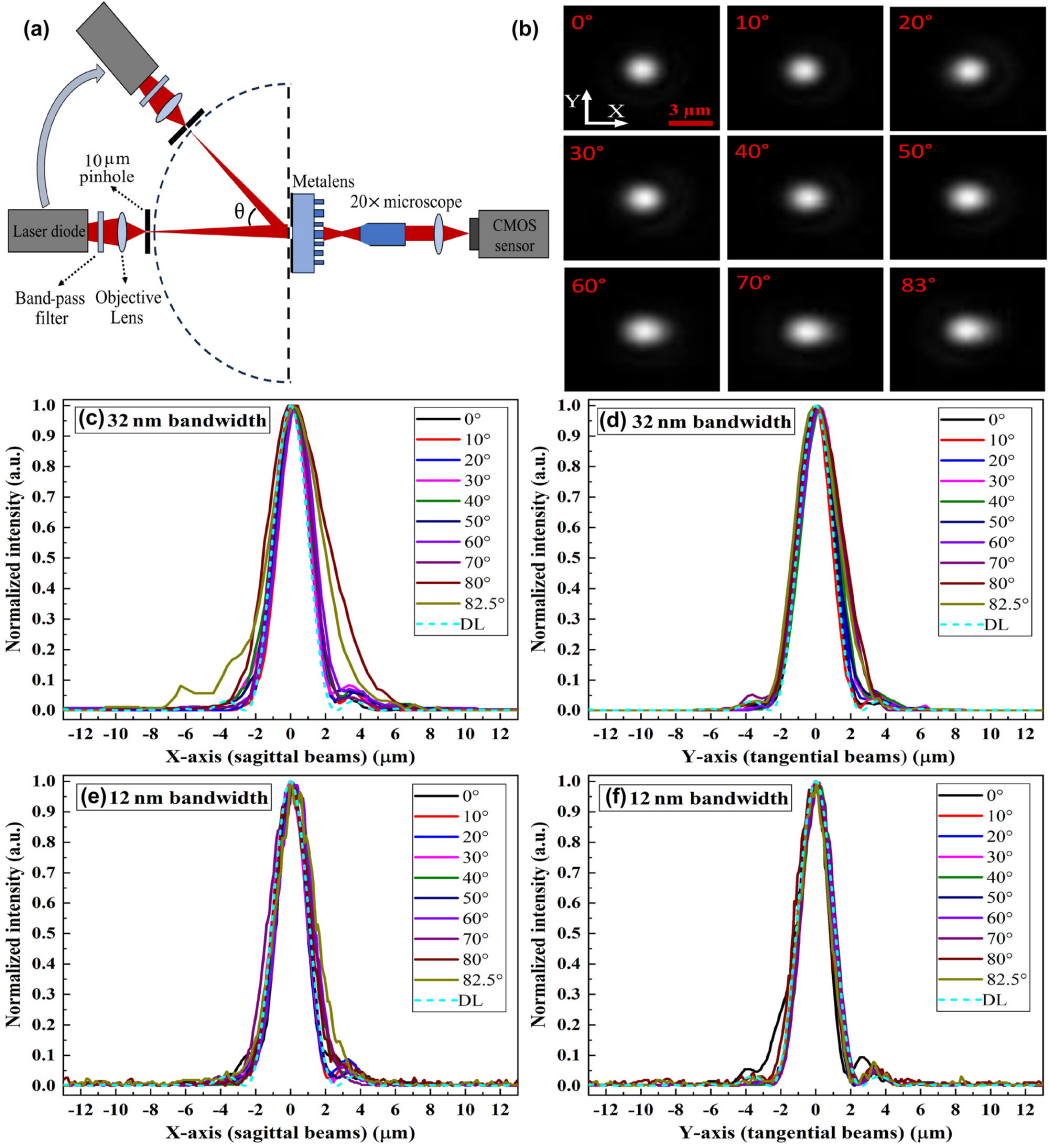
PSF characterization and analysis. (a) Optical setup for PSF characterization. (b) The captured focal spots at different AOIs, with a scale bar of 3 μm. (c) Sagittal and (d) tangential PSF diagrams for the 32 nm bandwidth. (e) Sagittal and (f) tangential PSF diagrams for the 12 nm bandwidth. The dashed lines indicate diffraction-limited (DL) PSF.

To comprehensively evaluate the performance of our metalens-based capsule endoscopy system, we first conducted a series of rigorous optical characterizations on the metalens. Our analysis focused on key performance metrics, including PSF, MTF, SR, and FWHM. The characterization setup, illustrated in Figure 3(a), comprised several optical and mechanical components. This setup included a 20× Mitutoyo Plan Apo NIR Infinity Corrected Objective (*f* = 10 mm, NA = 0.4), an MT-4 Mitutoyo tube lens, an MT9J003 Onsemi CMOS sensor, a rotating stage, a 10 µm circular pinhole, a focusing lens with a 12 nm bandpass filter, and a 940 nm laser diode. The scanning angles for different FOVs in the *x*–*y* plane were obtained by rotating the stage, which held the 940 nm laser diode, focusing lens, and 10 µm pinhole. The 10-megapixel MT9J003 Onsemi CMOS sensor, with a small pixel size of 1.67 µm, provided sufficient sampling and FOV to accurately characterize the metalens. The PSF, MTF, SR, and FWHM diagrams are plotted and discussed using two configurations of optical source: (1) a laser diode with a spectral bandwidth of 32 nm and (2) a configuration using a filter to reduce the spectral bandwidth of the source to 12 nm.

**Figure 4:**
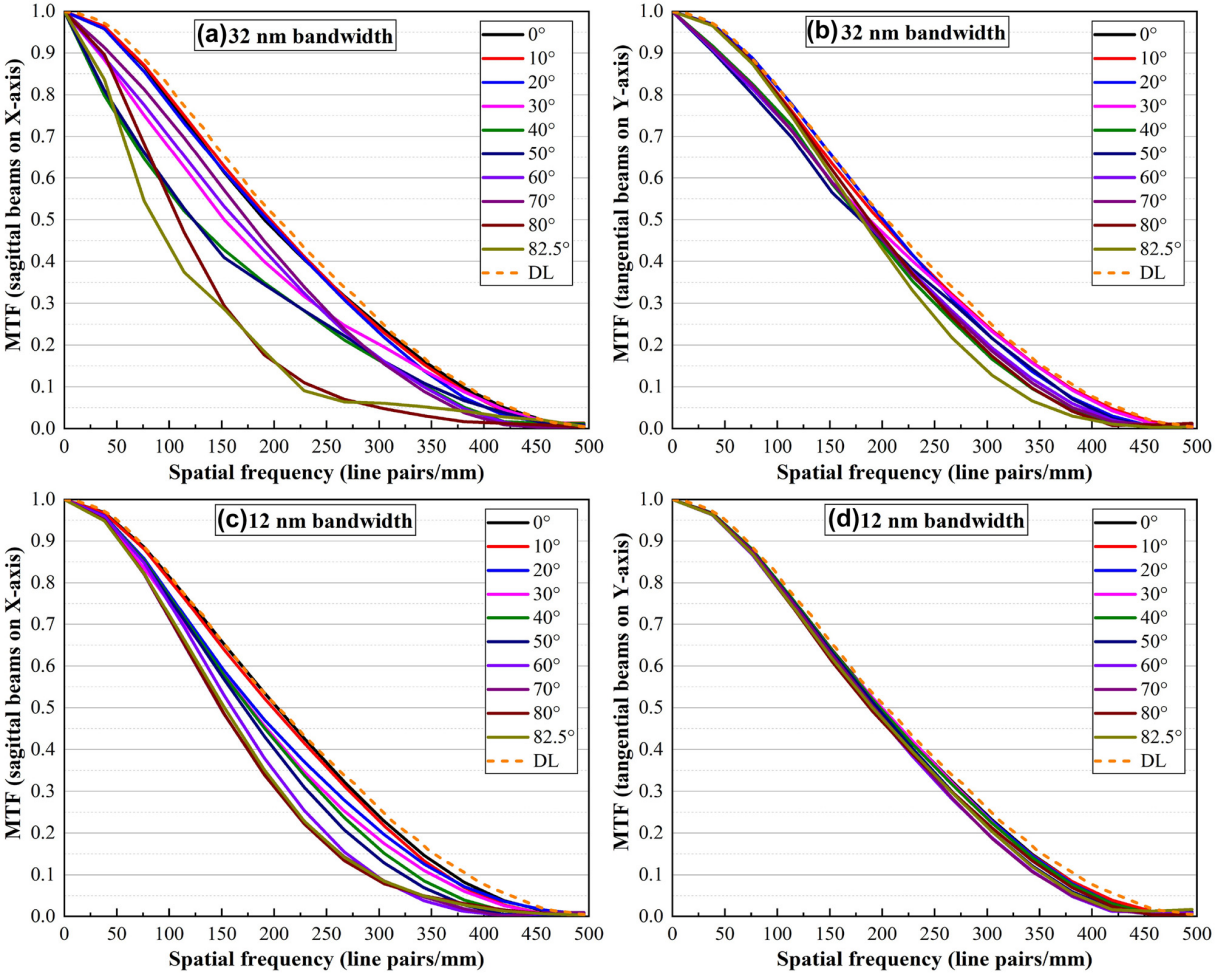
MTF diagrams at different AOIs for 32 nm bandwidth: (a) sagittal beams and (b) tangential beams, and for 12 nm: (c) sagittal beams and (d) tangential beams. The orange dashed lines indicate the diffraction-limited MTF.

Figure 3(b) illustrates the intensity distribution at the focal position for different angles of incidence (AOI) with a 32 nm spectral bandwidth. A comparison between the experimentally measured intensity distributions in Figure 3(b) and the simulated far-field intensity distribution in Figure S3 demonstrates that the experimental results closely match the FDTD simulations. The sagittal and tangential PSF diagrams at different FOVs are presented in Figure 3(c)–(f). These diagrams can be compared with the diffraction-limited diagram indicated by the dashed lines, demonstrating that both the sagittal and tangential diagrams closely approach the diffraction limit. The PSF results for the 32 nm bandwidth are worse than those for the 12 nm bandwidth, particularly at large angles where lateral chromatic aberration becomes evident. This is attributed to the design of the nano-pillars, which are optimized for a narrow band spectrum. Recently, advances in machine learning for disease diagnosis have increased the demand for high-quality images to diagnose a broader range of diseases and improve diagnostic accuracy. To achieve these objectives, it is essential to obtain high-contrast and high-resolution images across the entire FOV. The MTF diagrams of sagittal and tangential beams are presented in Figure 4 for two bandwidths, which are compatible with OpticsStudio analysis (Figure S1(b) and (c)) and FDTD results (Figure S3(b)). Figure 4 depicts an average MTF of 0.2 at 250 lp/mm with a 32 nm bandwidth, which improves to 0.3 at 250 lp/mm when using a 12 nm bandpass filter. The MTF results of the metalens demonstrate its ability to provide the high resolution and contrast necessary for capsule endoscopy. The comparison of experimental results with the MTF diagrams of a traditional bulky lens endoscope (Figure S6(e) and (f)) illustrates that the nano-optic endoscope provides higher resolution.

**Figure 5:**
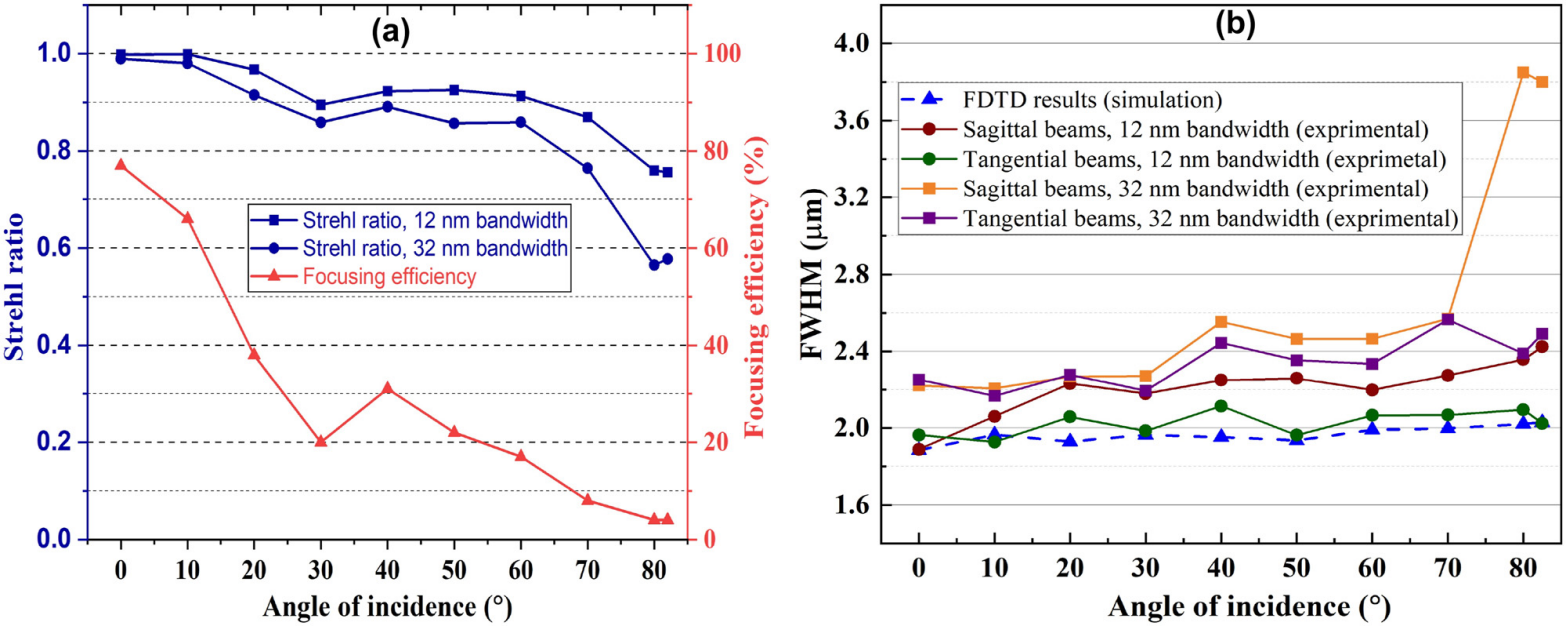
Focusing efficiency, Strehl ratio, and FWHM analysis for different AOIs. (a) The focusing efficiency and SR for two bandwidths. (b) Experimentally measured FWHM (solid lines) for tangential and sagittal beams compared with the simulated FWHM (blue dashed line).

The focusing efficiency can be defined as the ratio of the integral power at the focal spot to the incident optical power [43]. Achieving optimal focusing efficiency necessitates both high transmission and precise focusing capabilities. In this study, the focusing efficiency was calculated using a method similar to that in reference [19], by integrating the camera pixel values corresponding to the PSF intensity of the beam transmitted through the metalens at varying AOI, using the optical setup shown in Figure S4(a). These integrated values were then divided by the integration of the camera pixel values of the beam transmitted through a 260 μm pinhole, which corresponds to the aperture size. Throughout this measurement process, camera parameters, including gain and exposure time, were maintained at constant settings. The result of the focusing efficiency measurement is shown in Figure 5(a). The maximum focusing efficiency is 78 %, achieved at zero AOI, and decreases to 5 % as the AOI increases to 82.5°. Figure S4(c) illustrates that this decline is primarily due to the reduction in power transmission with increasing AOI. The power transmission across all AOIs is measured by the optical setup depicted in Figure S4(b). Simulated intensity peaks of focused beams using FDTD also demonstrate a decrease in intensity as the angle of incidence (AOI) increases, as depicted in Figure S4(d).

A suitable metric for imaging performance is the SR, which is measured as the ratio of the irradiance generated by the metalens to the irradiance produced by a diffraction-limited system with the same F-number (F/#). Blue diagrams in Figure 5(a) illustrate the SR metric for two bandwidths of 12 nm and 32 nm. Applying a 12 nm bandpass filter resulted in an average increase of 0.07 in the SR across various FOVs. The SR measured across different FOVs without the bandpass filter is over 0.56, while with the filter, it consistently exceeds 0.75. This indicates near diffraction-limited performance across the entire 165° FOV.

The FWHM for two bandwidths, one using a narrow bandpass filter and one without, are plotted in Figure 5(b) to compare the spot size of sagittal and tangential beams with the FDTD simulation results. The diffraction-limited FWHM of a zero-angle beam is 1.88 µm. The fabricated metalens exhibits FWHM values that are close to those predicted by the FDTD simulations. Additionally, employing a bandpass filter reduced the spectral bandwidth, resulting in a 9 % decrease in the FWHM. The difference in SR and FWHM parameters between the two bandwidths increases at larger AOIs due to lateral chromatic aberration, as previously discussed.

A comprehensive analysis of the traditional bulky lens endoscope camera, including focal spot distribution, FWHM, PSF, and MTF, is discussed in Supporting Information S4. The comparison between the nano-optic capsule endoscope, which utilizes metalens technology, and the traditional bulky lens endoscope reveals that the nano-optic capsule endoscope significantly better controls off-axis aberrations at large AOIs while also benefiting from a more compact size.

**Figure 6:**
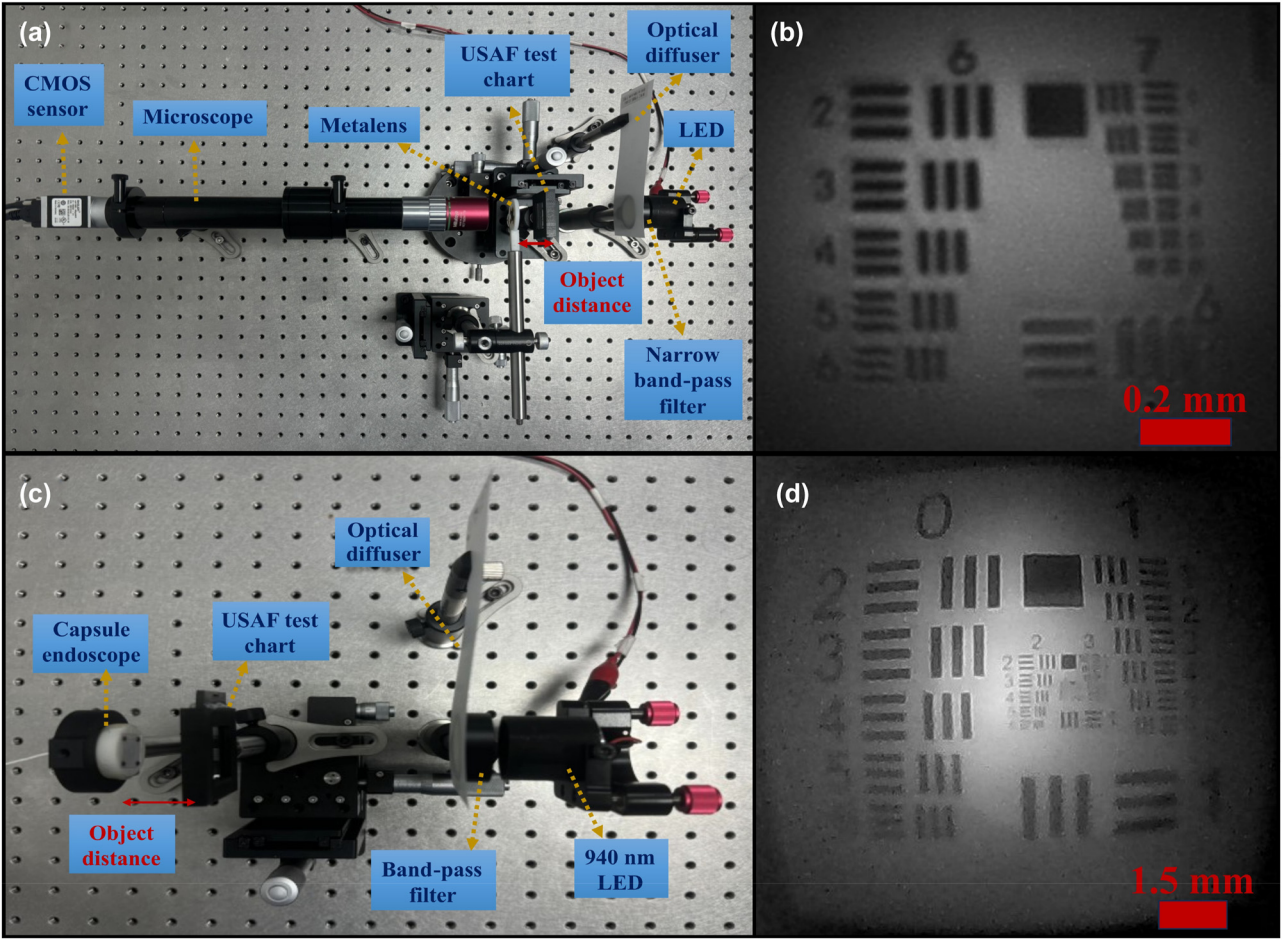
USAF 1951 resolution test chart. (a) Optical setup utilizing a 20× microscope and (b) its corresponding image test result. (c) Experimental image test setup for the capsule endoscope and (d) the resultant image.

Figure 6 illustrates the experimental setup and imaging resolution results using a USAF resolution test chart. Two configurations were employed in the study. Initially, a 20× microscope was utilized to capture the image. This configuration facilitated easy fine alignment. As depicted in Figure 6(b), the resolution achieved corresponds to group 7, element 2, which translates to 143.7 lp/mm, indicating that a line-width of 3.48 μm can be resolved. Subsequently, the test was conducted using an assembled capsule endoscope. Aligning the sensor with the metalens in this setup posed a significant challenge. Figure 6(d) presents the imaging results of the assembled capsule endoscope, resolving group 2, element 6.

It can be noted that by comparing two spectral bandwidths of 32 nm and 12 nm, the narrower bandwidth can provide a higher contrast of about 10 %. However, the 32 nm bandwidth of commercial NIR LEDs still provides high image quality.

**Figure 7:**
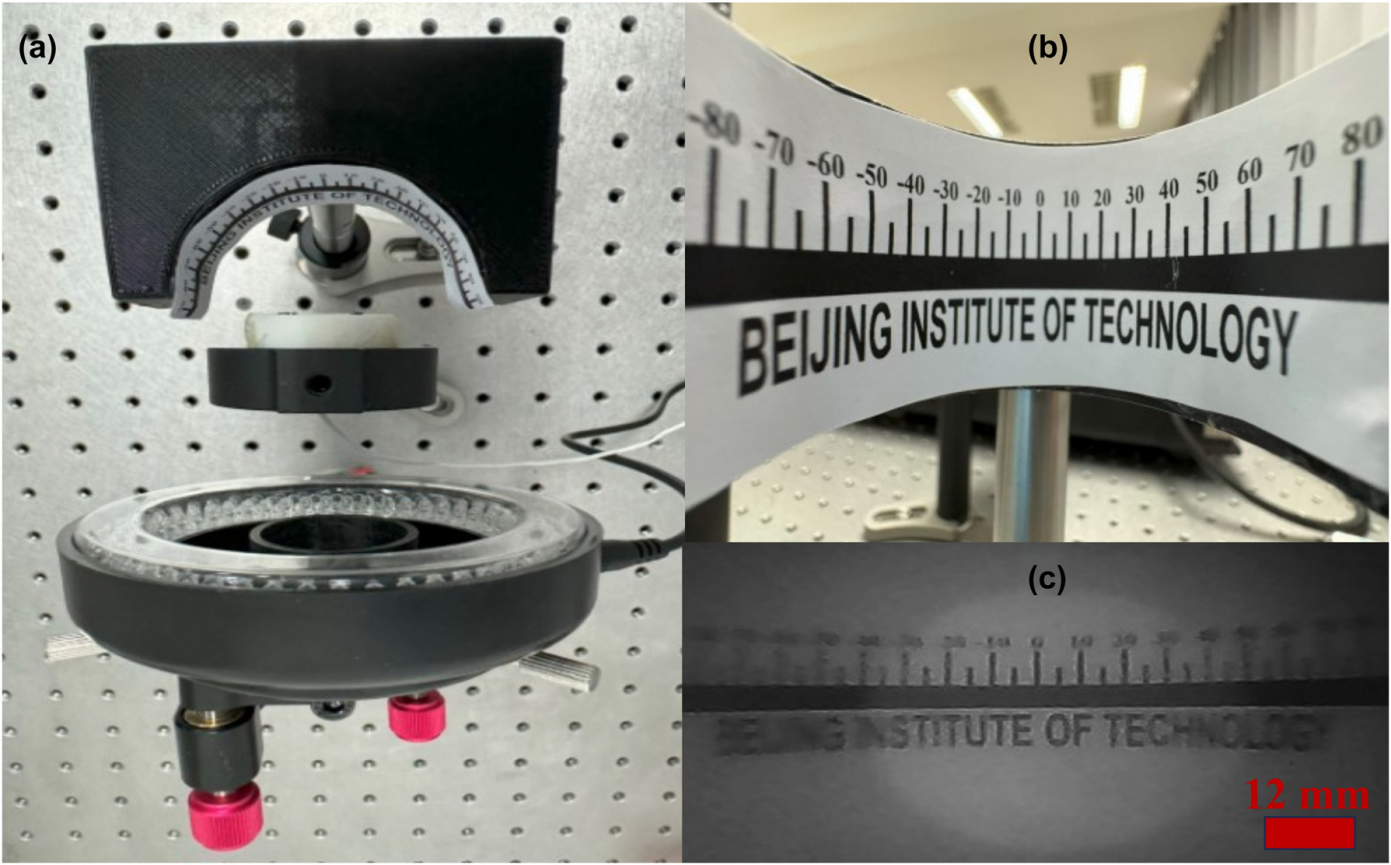
Actual test of the capsule endoscope. (a) Close-up view of the experimental setup. (b) A curved protractor labeled “Beijing Institute of Technology.” (c) The real image was captured using the capsule endoscope.

Figure 7 demonstrates the actual imaging result captured through the nano-optic capsule endoscope. The text “Beijing Institute of Technology” is visible, showing how the metalens focuses and projects a wide FOV object. The clarity and resolution of the text provide insights into the performance and quality of the wide FOV metalens. The image contrast is lower than predicted from other analyses such as PSF, MTF, SR, and FWHM, which were measured using a 20× microscope. This discrepancy arises from several issues, the primary one being the alignment of the focal distance. While precise alignment was achievable with the 20× microscope using a high-precision linear stage, it was more challenging for the capsule endoscope due to its small focal distance (755 µm), which is assembled using screws. This problem can be mitigated by either increasing the focal distance or employing robotic adjustment. Furthermore, distortion at high FOVs reduced contrast, which is inevitable in wide-FOV optical systems. However, it can be improved by postprocessing algorithms [44].

**Figure 8:**
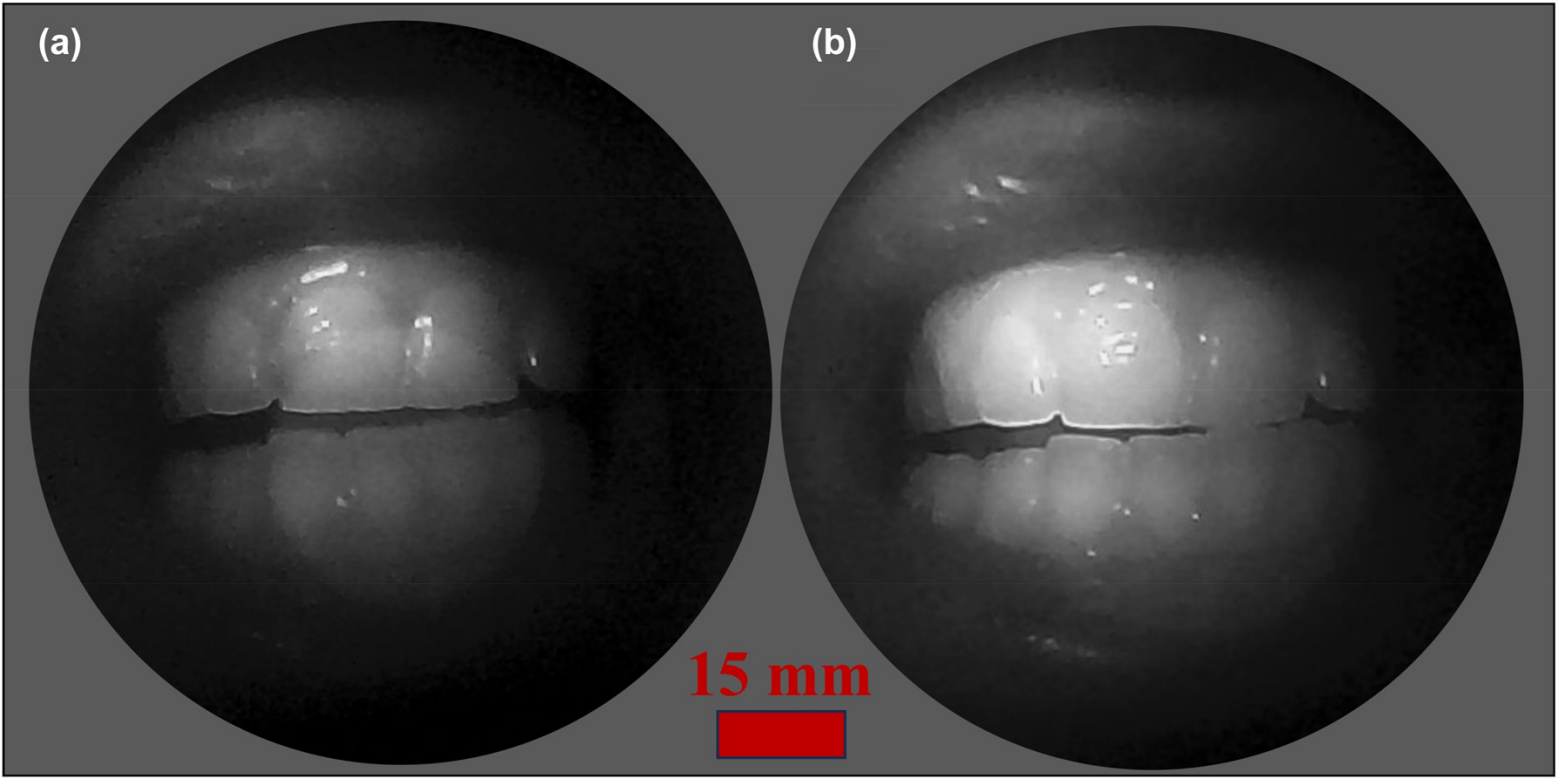
Images of teeth captured by nano-optic capsule endoscope at object distances of (a) 30 mm and (b) 15 mm.

Figure 8(a) and (b) present the narrow band images captured by the capsule endoscope device at object distances of 30 and 15 mm, respectively. The central focus of the image is on the teeth and oral cavity, which are illuminated and captured with high contrast and clarity. The image quality can be significantly improved by applying uniform illumination. This image demonstrates the capability of the metalens to capture detailed anatomical structures, essential for diagnostic purposes in medical applications. A central bright circle flare artifact is observed in the images captured by the assembled endoscope capsule, as depicted in Figures 7 and 8. This artifact was absent in the microscope image test (Figure 6(a)) and was not prominent at near object distances (5–20 mm), as was not detected in Figure 8(b). The artifact is identified as an out-of-focus ghost image of the iris. A similar issue has been reported in related wide FOV metalens [19], [44], [45]. This flare effect can be mitigated through postimage processing techniques. The images reconstructed via either the UNet and Knowledge Distillation (KD) paradigm, or a transform-based neural network trained with the KD paradigm, effectively mitigate the central bright speckle artifact [45]. The implementation of neural network algorithms for image recovery yields a substantial improvement in the contrast of images captured by the proposed metalens system.

The metalens measures 1.4 mm in length and 1.58 mm in diameter. In comparison, the traditional bulky lens discussed in Supporting Information S4 has a length of 3.1 mm and a diameter of 3 mm. This comparison highlights the superior compactness of the metalens, accompanied by enhanced FOV and image quality. Further device length reduction is possible by integrating metalens by the protective window of the CMOS sensor. This reduction in size enables the design of a more compact capsule, enhancing patient comfort compared to traditional capsules [7]. Conventional capsules typically have a length and diameter of approximately 25 and 11 mm, respectively. By incorporating the metalens into capsule endoscopy, the diameter and length can be reduced to about 15 and 7 mm, respectively, while simultaneously delivering higher performance.

To validate a proof-of-concept application, we acquired video footage using the nano-optic capsule endoscope. The recording is available in the Supporting Information Video. The high-definition video presents narrow bandpass image visualization of the oral cavity using a nano-optic capsule endoscope, featuring a 165° FOV and 30 frames per second in the NIR region. This footage underscores the metalens’s capability to maintain focus across varying depths, providing both detailed close-ups and expansive views of the oral anatomy. Additionally, the central bright circle flare artifact also appears in the video, which has been discussed before. The system’s high-resolution imagery and advanced functionalities demonstrate significant potential for enhancing diagnostic accuracy in gastrointestinal examinations.

## 4 Conclusions

In this paper, the application of a wide FOV metalens for a compact capsule endoscopy device is demonstrated. The advantages of metalenses, particularly their compactness and high image quality, make them well-suited for this application. Experimental characterization of the metalens validated our theoretical design and demonstrated NBI in the NIR region. Our results indicate an average MTF of 0.2 at 250 lp/mm with a spectral bandwidth of 32 nm. By using a narrow-bandpass filter to reduce the spectral width to 12 nm, the MTF can be increased to 0.3 at 250 lp/mm. A comparative analysis between conventional endoscope cameras with a 108° FOV and metalens technology demonstrates that metalenses offer a more compact design and superior mitigation of off-axis aberrations. The compactness, high optical performance, and multifunctionality of metalenses render them particularly well-suited for medical applications, especially in the context of capsule endoscopy. Additionally, this design can be adapted for other wavebands in endoscopy applications.

## Supplementary Material

Supplementary Material Details

Supplementary Material Details
