# Supplementary material for: Wide FOV metalens for near-infrared capsule endoscopy: advancing compact medical imaging
Source: Nanophotonics. 2024 Oct 17;13(24):4417–28. doi: 10.1515/nanoph-2024-0393 (PMC11636453; doi:10.1515/nanoph-2024-0393)
Supplement: Supplementary file 1 — Supplementary Material Details [file j_nanoph-2024-0393_suppl_001.docx]

**Supporting information**

**Wide FOV Metalens for Near-Infrared Capsule Endoscopy: Advancing Compact Medical Imaging**

Mojtaba Moghaddasi,^1^ Erik Edilson Perez Coca,^2^ Danni Ye,^3^ Diego Alejandro Flores,^4^ Xudong Wu, ^1^ Abdul Jalal, ^1^ Ziming Ren, ^1^ Fahimeh Abrinaei, ^5,6^ and Bin Hu,^1,*^

^1^ Beijing Engineering Research Center for Mixed Reality, School of Optics and Photonics, Beijing Institute of Technology, Beijing 100081, China

^2^ Department of Mechatronics, Beijing Institute of Technology, Beijing 100081, China

^3^ School of Chinese Materia Medica, Beijing University of Chinese Medicine, Beijing 102488, China

^4^ School of Computer Science and Technology, Beijing Institute of Technology, Beijing 100081, China

^5^ Department of Physics, Central Tehran Branch, Islamic Azad University, Tehran, Iran

^6^ Department of Physics, East Tehran Branch, Islamic Azad University, Tehran, Iran

**S1. Zemax OpticStudio analysis**

The optical design is done by Zemax OpticStudio and the physical optics propagation method is applied to optimize the phase distribution. Figure S1(a) illustrates the spot size analysis across all FOVs. The geometric spot and RMS radius (root mean square radius) are less than 2.4 and 0.9 µm, respectively. The MTF diagrams of the sagittal and tangential beams are plotted in Figure S1(b, c), respectively. The diagrams indicate the diffraction-limited phase distribution over all FOVs.


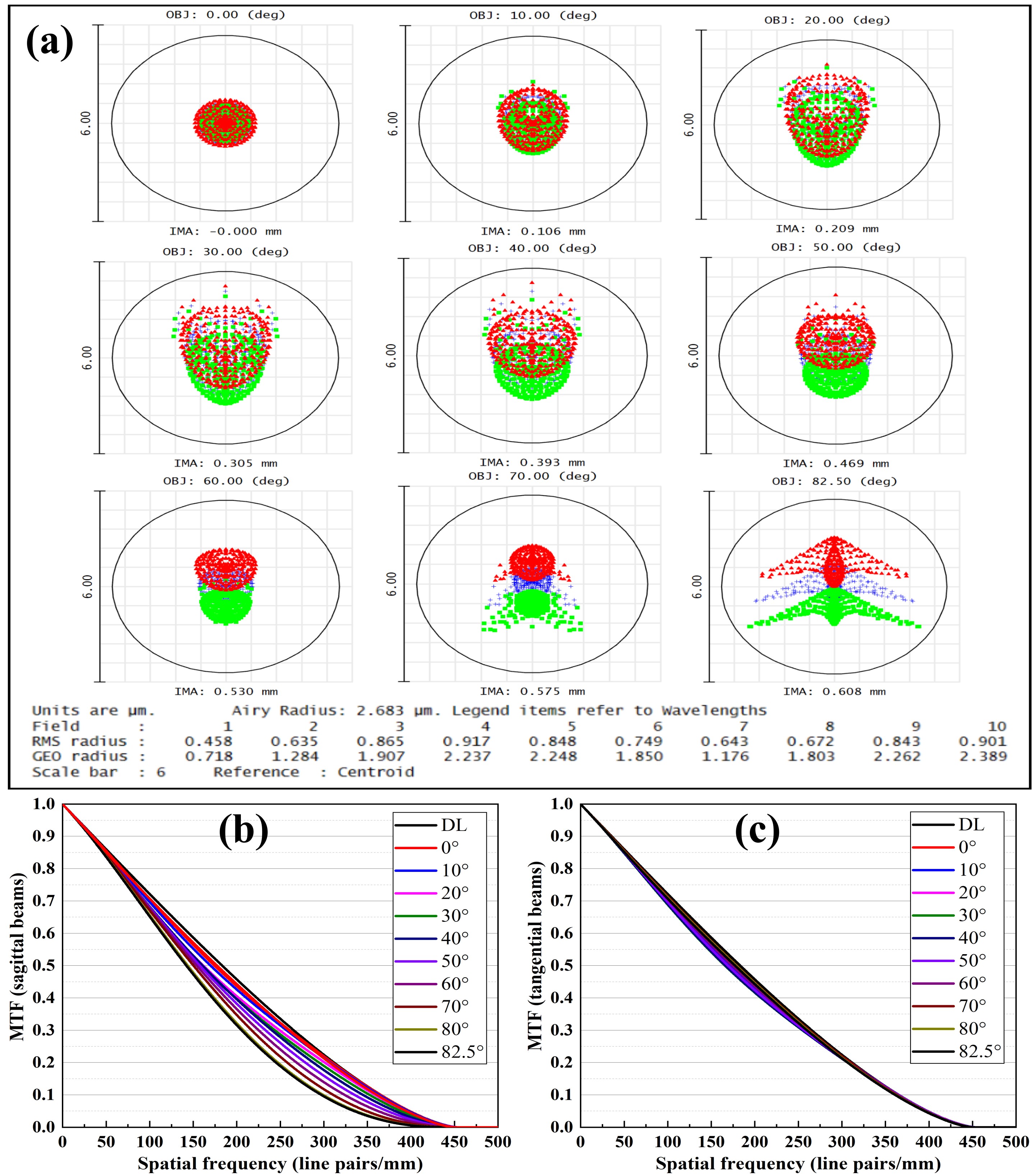


**Figure S1**. (a) Spot diagram analysis by ray tracing method of Zemax OpticsStudio. The red, blue, and green correspond to 939 nm, 940 nm, and 941 nm, respectively. The circles around each spot size show the Airy disc with a diameter of 5.4 μm. Polychromatic MTF diagram of (b) sagittal and (c) tangential beams.

**S2.** **Lumerical FDTD analysis**

Figure S2 displays the far-field intensity distribution of a metalens as determined by FDTD simulation for incident angles ranging from 0° to 82.5° in the x–z plane at the focal position.


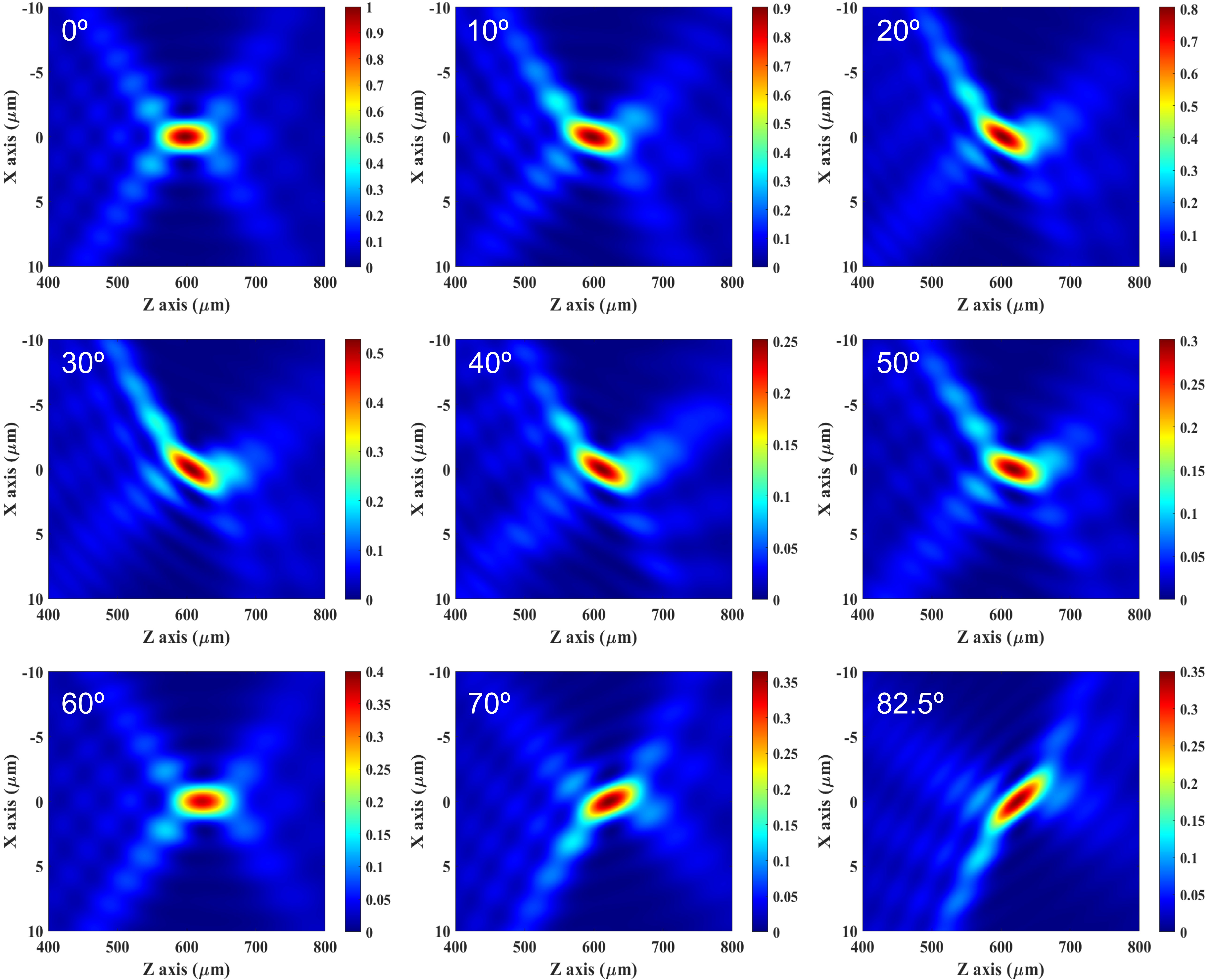


**Figure S2.** Far-field intensity distribution determined by FDTD simulation for incident angles ranging from 0° to 82.5° in the x–z plane at the focal position.

Figure S3 presents the sagittal MTF and PSF diagrams of the designed metalens at various AOIs. The MTF diagram indicates diffraction-limited (DL) performance across all FOVs. The PSF diagram demonstrates a decrease in intensity peak as the AOI increases. Although this results in darker edges of the field, it can be mitigated by using LED illumination in a circular shape, which brightens the edges more than the center.


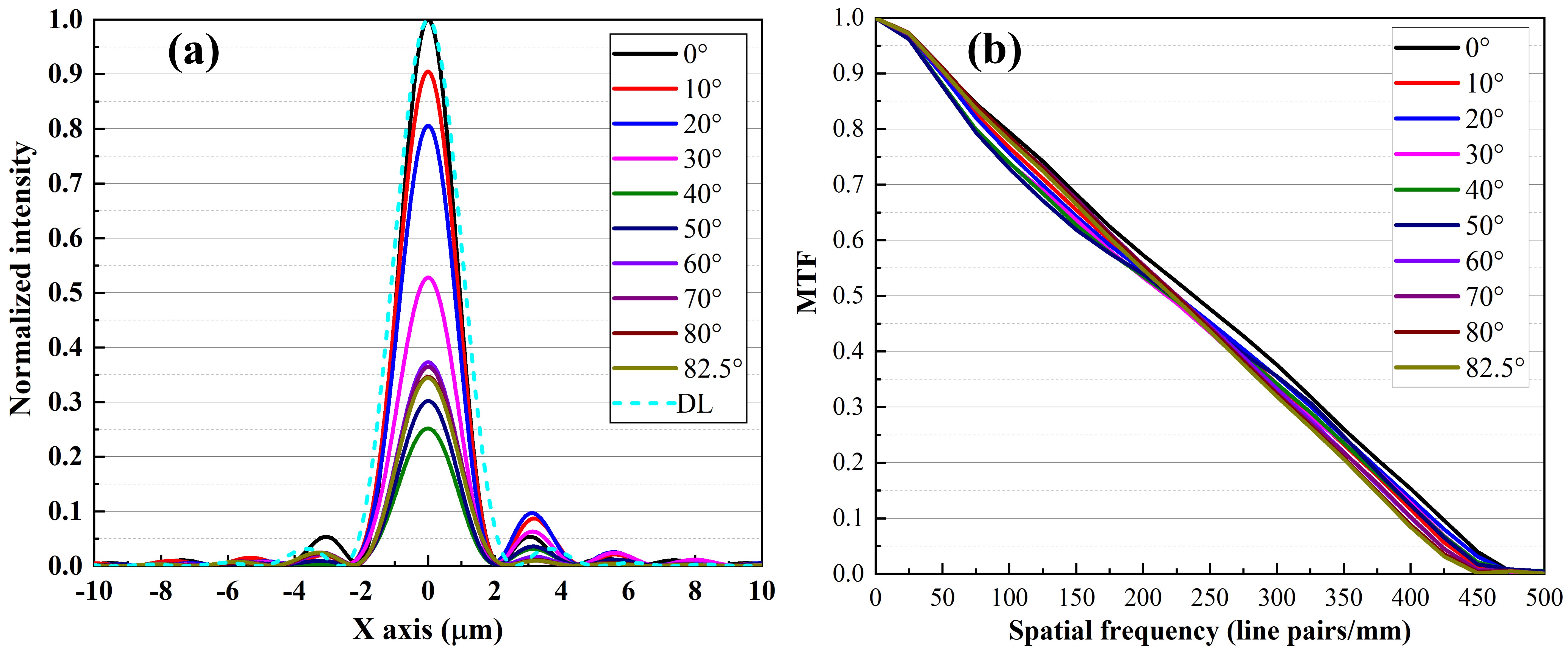


**Figure S3**. Sagittal PSF diagrams and (b) sagittal MTF diagrams for different AOIs simulated by FDTD simulation.

**S3.** **Focusing efficiency and transmission measurement**

The focusing efficiency was evaluated using the setup depicted in Figure S4(a). This setup closely resembles the one employed for measuring the PSF, but it utilizes a fiber-coupled laser diode (labeled as LD in Figures) with high power stability. This modification helps minimize errors arising from laser power fluctuations, ensuring more accurate efficiency measurements. The focusing efficiency data is presented in Figure 5(a), which reveals a decrease in power transmission at high AOIs. To further characterize the metalens performance, the power transmission was measured using the setup illustrated in Figure S4(b). The resulting power transmission data is plotted in Figure S4(c).

A 940 nm NIR laser diode was placed on a rotation stage, while the power meter and metalens remained in fixed positions. The transmission data were recorded over all FOVs and illustrated in Figure S4(d). Interestingly, the observed angle-dependent behavior was also replicated in the FDTD simulation results. Although metalens experienced reduced transmission and consequently lower focusing efficiency at high AOIs, this drawback can be mitigated by increasing the illumination power of the LEDs positioned at the corners.


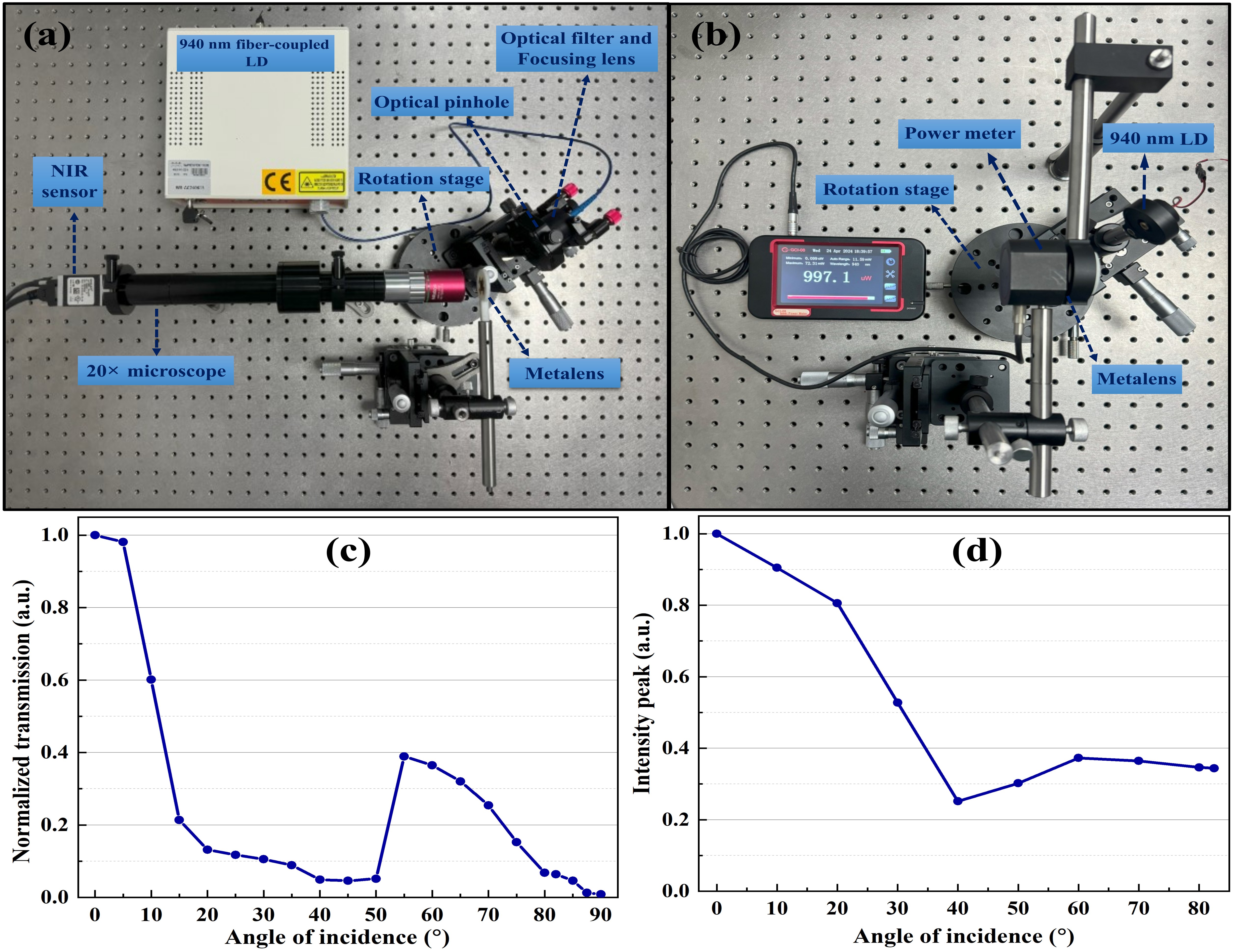


**Figure S4.** (a) Focusing efficiency measurement setup. (b) Transmission measurement setup. (c) Experimentally measured normalized transmission of the metalens. (d) Simulated intensity peak of focused beams.

**S4.** **Investigating the performance of a traditional endoscope for comparison**

Figure S5(a) presents a sectional view depicting the capsule endoscope during its assembly phase. The OV9734 sensor is securely affixed within the body of the capsule endoscope. For comparative evaluation, specifications of the compact 108° optics endoscopy camera M73512-G08 manufactured by Shenzhen Yong Onstar Photoelectric Co., LTD are assessed. as illustrated in Figure S5(b). The endoscopy camera features a focal length of 780 mm, an f-number of 4.6, and physical dimensions measuring 12 mm in length and 3.5 mm in diameter. Figure S6 shows diagrams detailing focal spots, FWHM, PSF, and MTF at various AOIs recorded using the mentioned traditional endoscope.


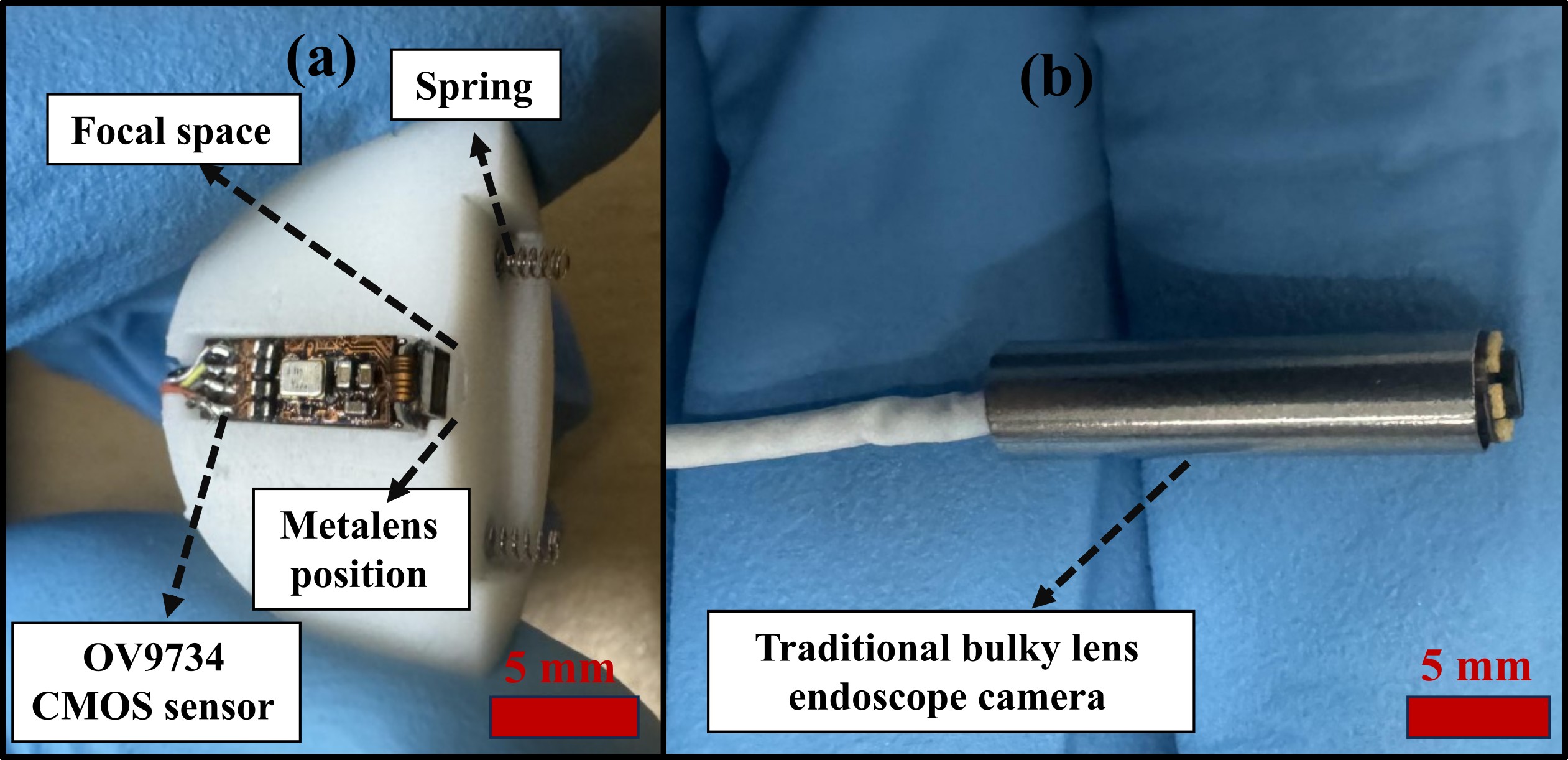


**Figure S5**. (a) A sectional view depicting the capsule endoscope structure. (b) Traditional bulky lens endoscopy camera.


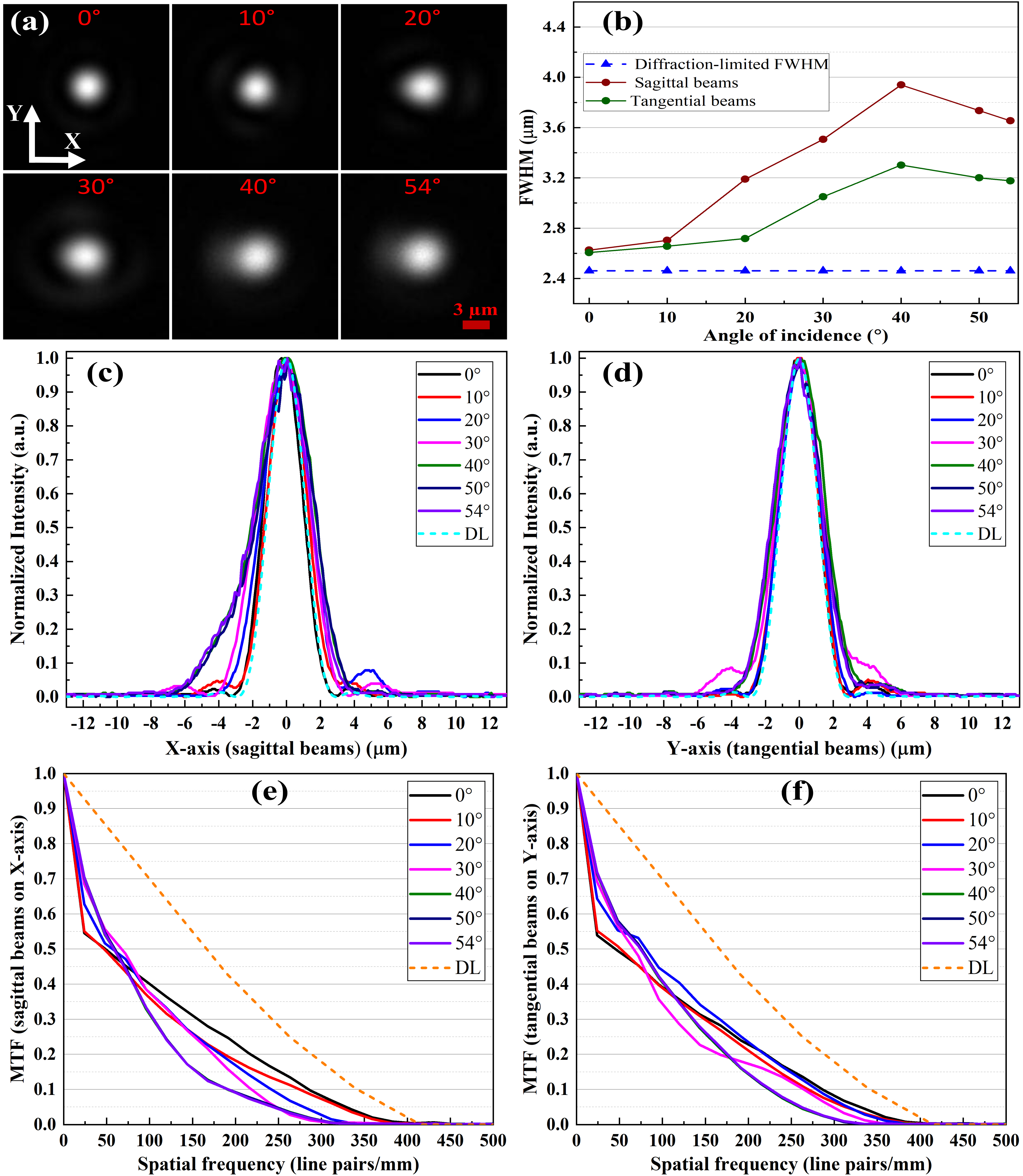


**Figure S6**. The results were recorded using a bulky lens endoscopy camera: (a) The captured focal spots, with a scale bar of 3 μm. (b) Experimentally measured FWHM for tangential and sagittal beam profiles (solid lines). (c) Sagittal and (d) tangential PSF diagrams. The dashed lines indicate DL PSF. MTF diagrams for (e) sagittal and (f) tangential. The orange dashed lines indicate DL MTF.

The characterization of the PSF diagram utilized a similar test setup as depicted in Figure S4(a), with a 532 nm laser source. According to Figure S6(a), larger incidence angles result in larger focal spots, particularly at the edges, due to off-axis optical aberrations. The PSF diagrams for the bulky optics camera (Figures S6(c, d)) exhibit significant variation and potential asymmetry at the periphery, whereas the nano-optic capsule endoscope maintains more uniform PSFs (Figures 3(c-f)), optimized for close-range imaging within the human body.

According to Figure S6(b), sagittal and tangential FWHM diagrams at small AOIs (0-10°) closely approached the diffraction-limited FWHM (2.46 µm). However, both sagittal and tangential FWHM values increase to 3.94 µm and 3.3 µm, respectively, at a 40° AOI. In contrast, as illustrated in Figure 5(b), the nano-optic capsule endoscope demonstrates reduced off-axis aberrations across its entire FOV. Additionally, the MTF diagrams in Figures S6(e, f) indicate that the bulky optics camera may experience a decline in MTF at higher spatial frequencies and larger incidence angles due to optical aberrations. Conversely, the nano-optic capsule endoscope preserves higher MTF values across a range of spatial frequencies and angles, tailored for high-contrast imaging of fine details necessary for medical diagnostics. The nano-optic capsule endoscope exhibits consistent optical performance across its entire FOV, which is crucial for diagnostic precision, whereas the 108° bulky optics camera, with its narrower FOV, exhibits reduced sharpness and resolution, especially towards the periphery.

The primary optical parameters of the nano-optic capsule endoscope are compared with those of three commercial capsule endoscopes in Table S1. The nano-optic capsule endoscope offered the highest FOV and the potential to be the most compact due to the compactness of the metalens. The current design also achieved the greatest depth of field. The functionality of metalenses enhances the practicality of this technology for capsule endoscopy devices; for instance, metalenses can be integrated with CMOS sensors or applied in polarization imaging within capsule endoscopy.

Table S1. A comparison of commercial capsule endoscopes with nano-optic capsule endoscope. N/A shows that the data is not available on the official website.

| Company | Nano-optics capsule endoscope | Olympus | Medtronic | Jinshan science and technology |
| --- | --- | --- | --- | --- |
| Model | This research | EC-S10 | PillCam SB3 | OMOM NC100 |
| FOV (º) | 165 | 160 | 156 | 160 |
| Diameter (mm) | <7 | 11 | 11 | 11.5 |
| Length (mm) | <15 | 26 | 26 | 31 |
| Depth of field (mm) | 5-100 | 0-20 | N/A | 0-35 |
| Contrast | 0.3 at 250 line pair/mm | N/A | N/A | Resolution of 8 line pair/mm |
| Future functionality | - Polarization imaging  - CMOS Sensor and metalens integration | N/A | N/A | N/A |
